# Trichostatin A ameliorates Alzheimer’s disease-related pathology and cognitive deficits by increasing albumin expression and Aβ clearance in APP/PS1 mice

**DOI:** 10.1186/s13195-020-00746-8

**Published:** 2021-01-04

**Authors:** Qiang Su, Tian Li, Pei-Feng He, Xue-Chun Lu, Qi Yu, Qi-Chao Gao, Zhao-Jun Wang, Mei-Na Wu, Dan Yang, Jin-Shun Qi

**Affiliations:** 1 Department of Physiology, Key Laboratory of Cellular Physiology, Ministry of Education, Shanxi Medical University, Taiyuan, 030001 Shanxi China; 2 Institute of Medical Data Sciences and School of Management, Shanxi Medical University, Taiyuan, 030001 Shanxi China; 3 Department of Hematology, the Second Medical Center & National Clinical Research Center for Geriatric Diseases, Chinese PLA General Hospital, Beijing, 100853 China

**Keywords:** Trichostatin A, APP/PS1 mice, Amyloid β clearance, Learning and memory, Albumin, Microglia

## Abstract

**Background:**

Alzheimer’s disease (AD) is an intractable neurodegenerative disorder in the elderly population, currently lacking a cure. Trichostatin A (TSA), a histone deacetylase inhibitor, showed some neuroprotective roles, but its pathology-improvement effects in AD are still uncertain, and the underlying mechanisms remain to be elucidated. The present study aims to examine the anti-AD effects of TSA, particularly investigating its underlying cellular and molecular mechanisms.

**Methods:**

Novel object recognition and Morris water maze tests were used to evaluate the memory-ameliorating effects of TSA in APP/PS1 transgenic mice. Immunofluorescence, Western blotting, Simoa assay, and transmission electron microscopy were utilized to examine the pathology-improvement effects of TSA. Microglial activity was assessed by Western blotting and transwell migration assay. Protein-protein interactions were analyzed by co-immunoprecipitation and LC-MS/MS.

**Results:**

TSA treatment not only reduced amyloid β (Aβ) plaques and soluble Aβ oligomers in the brain, but also effectively improved learning and memory behaviors of APP/PS1 mice. In vitro study suggested that the improvement of Aβ pathology by TSA was attributed to the enhancement of Aβ clearance, mainly by the phagocytosis of microglia, and the endocytosis and transport of microvascular endothelial cells. Notably, a meaningful discovery in the study was that TSA dramatically upregulated the expression level of albumin in cell culture, by which TSA inhibited Aβ aggregation and promoted the phagocytosis of Aβ oligomers.

**Conclusions:**

These findings provide a new insight into the pathogenesis of AD and suggest TSA as a novel promising candidate for the AD treatment.

## Background

Alzheimer’s disease (AD), a chronic neurodegenerative disease, is the most common cause of dementia in elderly individuals. According to World Alzheimer Report, dementia afflicts more than 47 million people worldwide, and this number will increase to 152 million by 2050 [1], while in which AD accounts for about 60–80% [2]. Unfortunately, there is still lack of efficient therapeutic drugs to reverse the progression of AD. It is well known that amyloid β (Aβ) accumulated in the brain is a key neuropathological hallmark of AD, leading to synaptic dysfunction and neuronal apoptosis [3, 4]. Aβ is produced from the sequential cleavage of amyloid precursor protein (APP) by β-secretase and γ-secretase [5, 6]. The imbalance between the production and clearance of Aβ has been considered as the major cause of excessive aggregation of neurotoxic Aβ in the brain [7, 8]. Since Aβ production and accumulation is an inevitable consequence in the progression of AD, targeting the clearance of Aβ in the brain of AD patients becomes a potential therapeutic strategy for AD. Intracellular Aβ can be cleared mainly through ubiquitin-proteasome pathway (UPP) and autophagy-lysosome pathway (ALP), while extracellular Aβ is degraded by multiple proteases such as neprilysin (NEP) and insulin-degrading enzyme (IDE) [9] and microglial phagocytosis [10]. Additionally, Aβ can be transported out of the brain and ultimately cleared by blood components, or some tissues and organs in periphery. Although many researchers reported their results on Aβ clearance, the detailed mechanisms underlying Aβ clearance are still not fully elucidated, and the drugs that could effectively remove Aβ remain to be further explored.

Drug repurposing referred to rediscovering a new indication for an existing drug [11]. In the current study, our group took such a strategy and found that trichostatin A (TSA) might be a potential therapeutic drug for AD. TSA, an inhibitor of histone deacetylase (HDAC) [12], is currently for anti-tumor therapy [13, 14]. Interestingly, the drug TSA showed neuroprotective effects in some experimental studies. For example, an animal study showed that short-term treatment of TSA by intraperitoneal injection improved conditioned fear memory and hippocampal CA3-CA1 long-term potentiation (LTP) and upregulated the level of hippocampal histone H4 acetylation in APP/PS1 mice, indicating that TSA could pass through the BBB and exert neuroprotective effects [15]. Another experiment showed that TSA increased the levels of plasma gelsolin and Aβ in AD transgenic mice, suggesting that plasma gelsolin might help to clear Aβ from the brain or other tissues [16]. Furthermore, it was found that TSA also increased gelsolin expression in the brain and prevented the formation of new amyloid deposits, but increased the size of existing plaques [17]. In addition, a recent study showed that TSA attenuated Aβ-induced cytotoxicity of SH-SY5Y cells by activating nuclear factor erythroid 2-related factor 2 (Nrf2) signaling [18]. However, there is still a lack of a systematic research on the effects of TSA in ameliorating cognitive deficits and pathological damage of APP/PS1 mice. Especially, the molecular mechanisms underlying the neuroprotection of TSA remain to be clarified. In the present study, we reported the ameliorative effects of chronic intraperitoneal (i.p.) injection of TSA on the short-term recognition memory and long-term spatial memory of APP/PS1 mice by using multiple behavioral tests. Furthermore, we examined the Aβ clearance effects of TSA in the brain and peripheral blood in APP/PS1 mice and the molecular mechanisms underlying the Aβ clearance by TSA.

## Methods

### Animals

Male APPswe/PS1dE9 (APP/PS1) heterozygous mice and wild-type (WT) littermates were used in the present in vivo study. The APP/PS1 mice expressed human APP with Swedish mutations (K595N/M596L) and human PS1 gene with deletion of exon 9. All mice were obtained from the Model Animal Research Center of Nanjing University (Nanjing, China) and genotyped through tail clips and subsequent PCR analysis of genomic DNA. Mice were housed in a temperature-regulated room under a 12 h light-12 h dark cycle, with free access to food and water. The 8-month-old mice (*n* = 44) were randomly divided into four groups: WT + vehicle (*n* = 11), WT + TSA (*n* = 11), APP/PS1 + vehicle (*n* = 11), and APP/PS1 + TSA (*n* = 11). Based on a previous study [15], TSA (T6270, TargetMol) was solubilized in 100% dimethylsulfoxide (DMSO) and then diluted with normal saline to final concentration of 0.2 mg/mL. TSA (2 mg/kg) or equivalent vehicle (solvent of TSA) was administered via i.p. injection daily for 30 days before behavioral experiments. The injection was continued during behavioral experiments and kept until 2 weeks after the end of behavioral experiment (see Fig. S1).

### Novel object recognition test

Twenty-four hours after the open field test, the mice were subjected to a novel object recognition test to assess the short-term recognition memory of mice. As described previously [19], mice were firstly placed into the open field with two identical objects (object 1 and object 2) for familiarization. After a 6 h retention interval, mice were returned to the same open field for test, but one familiar object had been replaced by a novel object with different color and shape. In the either phase, each mouse was placed in the apparatus facing the wall and allowed to freely explore the objects for 10 min. The apparatus was washed with 75% ethanol solution before each trial. The behavior of mice was recorded by a video tracking system and analyzed using SMART 3.0 software (RWD Life Science). Novel object recognition memory was calculated as the “novel object recognition index” (NOI) = (exploration time of novel object)/(exploration time of familiar and novel objects) × 100%.

### Morris water maze test

Morris water maze (MWM) test was used to assess the long-term spatial learning and memory ability of mice [20]. Briefly, MWM test was performed in a white circular tank (120 cm in diameter, 50 cm in height), filled with tap water (22 ± 3 °C) and divided into four equal virtual quadrants. Non-toxic white paint was added into the water until it became opaque. Some different geometric cues were mounted on the white curtain surrounding the tank. In the place navigation training phase (four 60-s trials/day for five consecutive days), a circular escape platform (10 cm in diameter) was placed in the center of a targeted quadrant, submerged 1 cm beneath the surface of the water. Each mouse was placed in the water facing the wall of the tank from different release positions and allowed to search for the escape platform. Once the mouse escaped onto the platform, it was allowed to remain on the platform for 5 s. The escape latency to climb onto the escape platform was recorded. If the mouse failed to find the platform within 60 s, it was guided to climb on the platform and remained on it for 30 s. On the 6th day of probe test, mice were allowed to swim freely in the water without the platform for 60 s. The percentage of time spent in the target quadrant and the number of platform crossing were measured. Mice that floated in the maze were not enrolled in the test. Lastly, a visible platform test was performed to detect the visual ability of mice. The swimming speed was also measured during the MWM test to exclude the mice with motor deficits. The swimming tracks were monitored and analyzed by a computer-based video tracking system (Ethovision software, Noldus Information Technology).

### Tissue processing and antibodies

After 2 weeks recovery from behavioral experiments, the mice in each group were randomly divided into two cohorts, one for immunofluorescence staining and the other for single molecule array (Simoa) and Western blot (WB). After deeply anesthetized with 5% chloralhydrate (0.007 mL/g, i.p.), mice blood was collected via cardiac puncture. Plasma was separated by centrifugation at 5000×*g* for 15 min at 4 °C and immediately stored at − 80 °C. For immunofluorescence staining, mice were perfused transcardially with 0.01 M phosphate buffer saline (PBS) and subsequently with 4% paraformaldehyde (PFA). Afterwards, the brains were isolated and successively immersed in 4% PFA (24 h), 15% sucrose (24 h), and then 30% sucrose (48 h). Serial coronal sections (30 μm thick) were cut using a cryomicrotome (CM1950, Leica) and mounted on polylysine-coated slides. For Simoa and WB, mice were perfused through the hearts with 30 ml saline solution (0.9%) and rapidly decapitated, and then the brains were immediately removed. The hippocampus tissues were carefully dissected out on ice and immediately frozen in liquid nitrogen and then kept in a − 80 °C freezer.

The primary antibodies used in the study include the following: 6E10 (803015, Biolegend), D54D2 (8243, Cell Signaling), anti-LC3B (3868, Cell Signaling), anti-Iba 1 (019-19741, Wako), anti-APP-CTF (A8717, Sigma-Aldrich), anti-IDE (ab133561, Abcam), anti-p62 (ab109012, Abcam), anti-NEP (ab81688, Abcam), anti-ubiquitin (ab134953, Abcam), anti-BACE1 (ab2077, Abcam), anti-LRP1 (ab92544, Abcam), anti-Beclin 1 (ab207612, Abcam), anti-albumin (ab207327, Abcam), anti-ace-Histone H4 (Ac-H4) (ab177790, Abcam), and anti-β-actin (D191047, Sangon Biotech). The secondary antibodies include goat anti-rabbit IgG-HRP conjugate (BA1054, BOSTER), goat anti-mouse IgG-HRP conjugate (BA1050, BOSTER), goat anti-mouse IgG-Cy3 (BA1031, BOSTER), and goat anti-rabbit IgG-Cy3 (BA1032, BOSTER).

### Immunofluorescence and Thioflavin S (ThioS) staining

Brain slices were washed in PBS, permeabilized for 15 min by shaking at room temperature with PBS containing 0.5% Triton X-100, rinsed in PBS, and then blocked with 5% bovine serum albumin (BSA; AR1006, BOSTER) in PBS for 1 h at room temperature. Thereafter, sections were incubated overnight at 4 °C with primary antibody and then placed in a wet box containing a little water. After rinsing, sections were incubated with appropriate fluorescence-conjugated secondary antibodies at room temperature for 1 h. For ThioS staining, sections were stained with 0.05% ThioS (23059, AAT Bioquest) in 50% ethanol in dark for 8 min at room temperature, followed by two rinsing in 80% ethanol for 10 s each. Finally, DAPI (C0065, Solarbio) was used for nuclear staining. Slides were then sealed with the fluorescent mounting medium (AR1109, BOSTER). Immunofluorescence images were acquired using a fluorescent microscope (Olympus) and analyzed using the ImageJ software.

### Western blot (WB)

Hippocampal tissues were prepared as described above. Cells (see below) were washed twice with ice-cold PBS. The tissues and cells were homogenized in cold RIPA buffer (AR0102, BOSTER) containing a cocktail of PMSF (AR1179, BOSTER) and protein phosphatase inhibitor (AR1183, BOSTER). The homogenates were then centrifuged (16,000×*g*, 30 min, 4 °C), and the supernatants were collected. The total protein concentrations of the samples were measured by a BCA Protein Assay Kit (PC0020, Solarbio). Total protein for each sample was diluted 1:1 with loading buffer (AR0131, BOSTER) and boiled for 5 min at 95 °C. Equal amounts of total protein were separated by 12% or 15% SDS-PAGE (AR0138, BOSTER) and transferred to PVDF membranes (0.45 μm or 0.22 μm, Millipore). Following blocking with 5% BSA for 2 h at room temperature, the membranes were incubated with desired primary antibodies overnight at 4 °C, and then HRP-conjugated secondary antibody for another 2 h at room temperature. After several washes, the protein bands were developed with ECL Western Blot Detection kit (P0018FS, Beyotime) and detected using Azure c300 Chemiluminescent Western Blot Imaging System (Azure Biosystems). The band intensity was analyzed with AlphaView SA (Fluorchem FC3, ALPHA).

### Single molecule array (Simoa) assay for quantification of Aβ_40_ and Aβ_42_

The concentrations of Aβ_40_ and Aβ_42_ in the hippocampus and plasma were quantified using the commercially available Neurology 3-Plex Assay Kits (101995, Quanterix) on a Simoa platform (HD-1 Analyzer, Quanterix) according to the instructions by the manufacturer. Samples were diluted at a ratio of 1:4 and performed on a single run.

### Cell culture and reagent preparation

Murine hippocampal neuronal cell line (HT22) and murine neuroblastoma cell line (N2a) were respectively provided by the Department of Physiology and Department of Anatomy, Shanxi Medical University. Murine microglia cell line (BV2) was bought from the cell bank (Chinese Academy of Medical Sciences). Murine microvascular endothelial cell line (bEnd.3) was purchased from iCell Bioscience Inc. (China). Cells were cultured at 37 °C in a humidified 5% CO_2_ incubator and were maintained in DMEM media supplemented with 10% fetal bovine serum (FBS, ExCell) and antibiotics (100 U/mL penicillin and 100 μg/mL streptomycin). For morphological observation and Western blot, cells were seeded at a proper density into a 6-well plate and treated under the indicated conditions for 24 h, and then the protein extract was collected.

Recombinant Aβ_1–42_ peptide (ChinaPeptides) was dissolved in HFIP, sonicated for 30 min, aliquoted, lyophilized, and stored at − 80 °C until use. Lyophilized Aβ was resuspended in DMSO and diluted in PBS to the final concentration (10 μM and 20 μM). Albumin powder (30R-3304, Fitzgerald) was dissolved in PBS to the final concentrations of 10 μM and 20 μM. ITSA-1 (S8323, Selleck) powder was dissolved in DMSO and diluted in PBS to the final concentration of 50 μM.

### Transmission electron microscopy (TEM)

The morphological changes of Aβ aggregation in the presence or absence of albumin were characterized by TEM according to a previously published method [21]. Freshly dissolved Aβ (20 μM) was treated with albumin (20 μM) and incubated at 37 °C for 24 h with constant agitation. Glow discharged grids were treated with samples (5 μL) for 2 min at room temperature. The excess sample was removed with filter paper and then negatively stained with 1% phosphotungstic acid for 1 min. Phosphotungstic acid was blotted off and the grid was dried for 20 min at room temperature. Images were recorded on a JEM-1011 electron microscope (JEOL) at the voltage of 80 kV.

### Cell migration assay

Cell migration was detected using an 8-μm pore size culture insert (Corning) in a 24-well plate to assess the migration of BV2 cells. BV2 cells were suspended in standard medium and added in the upper chamber of the transwell system, and conditioned medium (contained Aβ (10 μM) or albumin (10 μM) or both) was added into the lower chamber. After 24 h incubation, cells migrating to the underside were fixed with 4% PFA and then stained with DAPI and counted at × 20 magnification with an immunofluorescence microscope (Olympus).

### Statistical analysis

SPSS 13.0 and SigmaPlot 12.3 were used for the statistical analysis. The escape latency and swimming speed in the MWM test were analyzed by two-way ANOVA with repeated measures followed by post hoc Tukey’s multiple comparison tests. The other data were assessed by unpaired Student’s *t* test or one-way ANOVA followed by the appropriate post hoc test. All data were presented as means ± standard error (SEM) with *p* < 0.05 considered as statistically significant.

## Results

### TSA ameliorated recognition memory and spatial memory of APP/PS1 mice

**Fig. 1:**
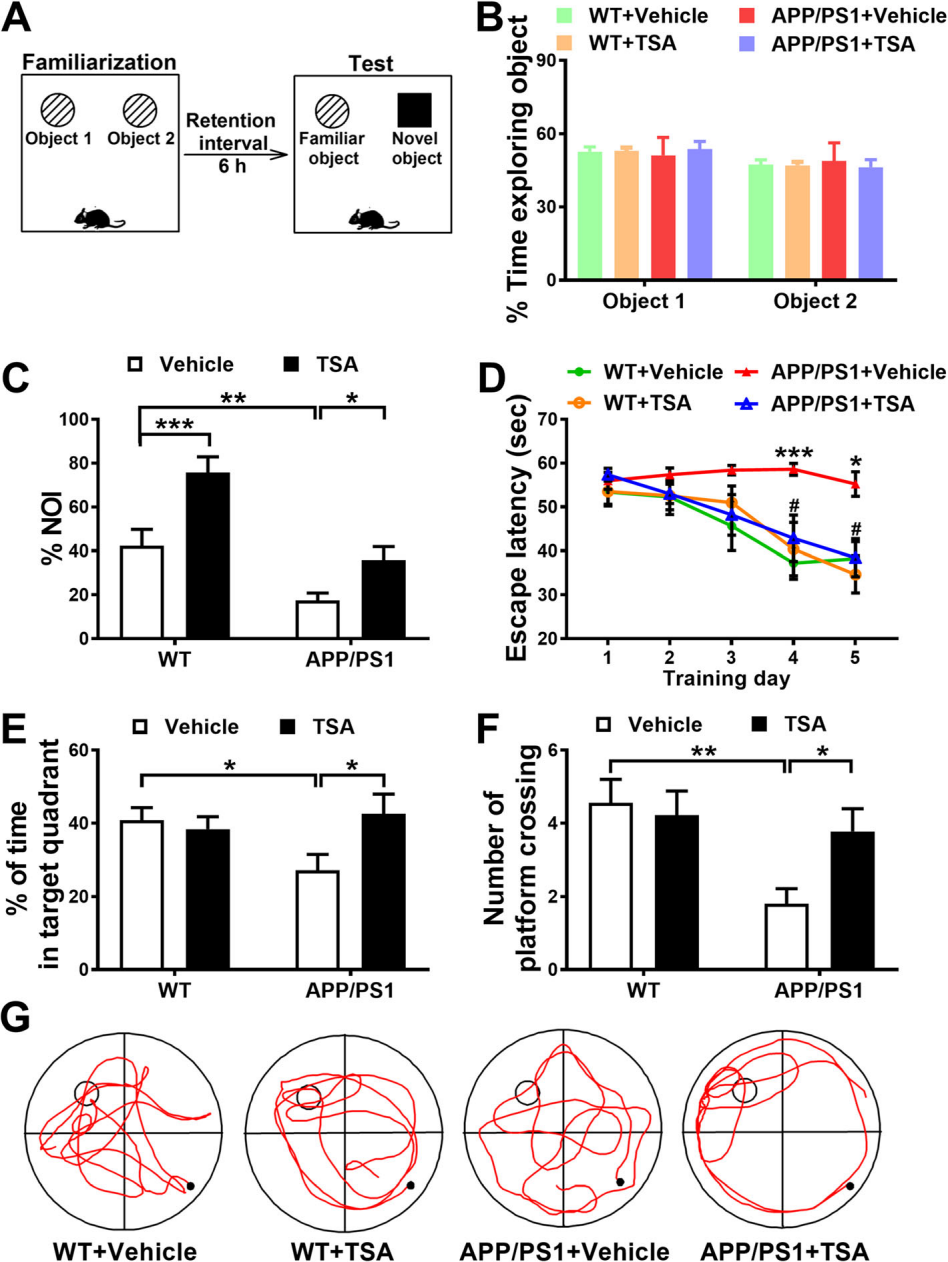
TSA treatment improved recognition memory and spatial learning and memory of APP/PS1 mice. **a** Schematic of the NORT. **b** The percentage of time exploring two identical objects (object 1 and object 2) during familiarization phase. **c** Novel object recognition index (NOI) during test phase in NORT. **d** Plots showing the changes of escape latency of mice to find the hidden platform during place navigation training phase. *vs WT + vehicle, #vs APP/PS1 + vehicle. **e** Histograms showing the percentage of swimming time of mice spent in the target quadrant. **f** Histograms showing the number of platform crossing in the probe test. **g** Representative trajectories of each group in the probe test. *n* = 9–10 in each group. *p < 0.05, **p < 0.01, ***p < 0.001 and ^#^*p* < 0.05

Novel object recognition test was firstly used to examine whether TSA could ameliorate recognition memory of APP/PS1 mice (Fig. 1a). In the familiarization phase (Fig. 1b), no difference in the exploration time for two identical objects was found among all groups (*p* > 0.05). During the test phase (Fig. 1c), the NOI in vehicle-treated APP/PS1 mice was significantly lower than that in vehicle-treated WT mice (*p* < 0.01), while TSA treatment basically reversed this decline in the APP/PS1 + TSA mice (*p* < 0.05). In addition, we noticed that TSA alone (WT + TSA) also markedly elevated NOI compared to vehicle-treated WT mice (*p* < 0.001). These results indicated that TSA treatment could mitigate the recognition memory deficits of APP/PS1 mice. Further, the MWM test was performed to evaluate the effects of TSA on the spatial learning and memory in APP/PS1 mice. As shown in Fig. 1d, the escape latency of vehicle-treated APP/PS1 mice was markedly longer on training day 4 (*p* < 0.001) and day 5 (*p* < 0.05), while TSA treatment significantly shorten the escape latency of APP/PS1 mice on day 4 (*p* < 0.05) and day 5 (*p* < 0.05), implying that TSA relieved the spatial learning deficit in APP/PS1 mice. In the probe test, the mice in APP/PS1 + vehicle group exhibited significant reductions in the percentage of swimming time in the target quadrant (*p* < 0.05) and the number of platform crossing (*p* < 0.01) compared with WT + vehicle group, whereas the reductions were reversed in APP/PS1 + TSA group (*p* < 0.05) (Fig. 1e, f), indicating that TSA effectively improved the spatial reference memory of APP/PS1 mice. In the visible platform test, the escape latency to reach the platform and the swimming speed of all mice did not show any significant difference among all groups (*p* > 0.05) (Fig. S2A and B), suggesting that the differences in performance among groups of mice in hidden platform and probe tests were not due to the changes in the visual acuity and swimming ability of mice.

### TSA reduced both Aβ plaques and Aβ oligomers in the hippocampus, as well as Aβ levels in the plasma of APP/PS1 mice

**Fig. 2:**
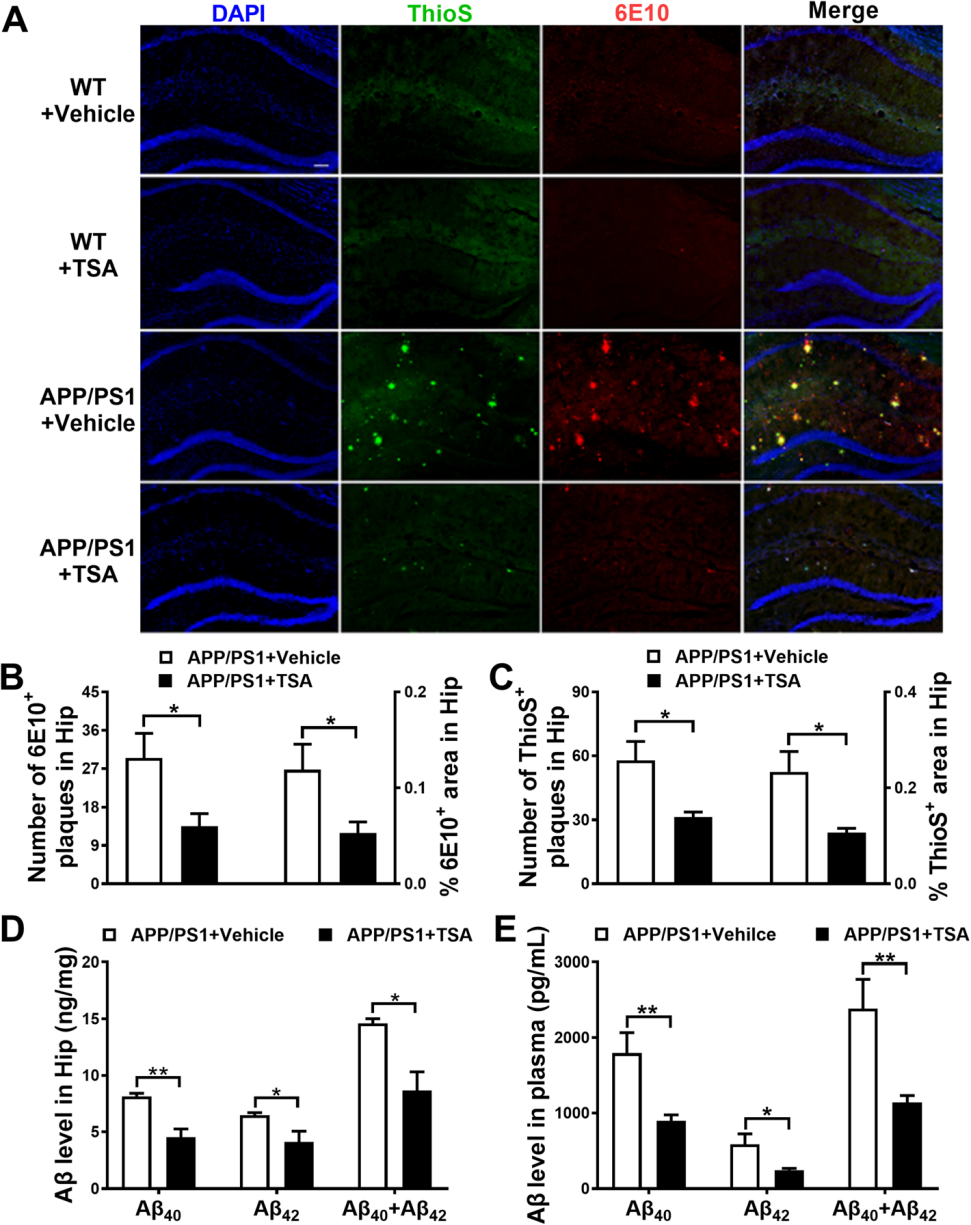
TSA treatment reduced Aβ level in the hippocampus and plasma of APP/PS1 mice. **a** Representative images showing Aβ plaques in the hippocampus of mice. Thioflavin S (ThioS), green; 6E10, red; DAPI, blue. Scale bar, 200 μm. **b** Histograms showing the number of 6E10-positive Aβ plaques in the hippocampus (Hip). **c** Histograms showing the percent area of ThioS-positive Aβ plaques in the hippocampus (Hip). *n* = 6 per group. **d**, **e** Simoa assay for the levels of Aβ_40_ and Aβ_42_ in the hippocampus (Hip) (**d**) and plasma (**e**) of APP/PS1 mice treated with vehicle or TSA. *n* = 4 per group. *p < 0.05 and **p < 0.01

Given that Aβ plaque is one important neuropathological hallmark in AD brains [22], we firstly evaluated whether TSA affected Aβ plaques in the hippocampus of APP/PS1 mice by Thioflavin S (ThioS) staining and Aβ immunostaining (6E10). Clearly, neither ThioS- nor 6E10-positive plaque was found in the WT mice (Fig. 2a), supporting the results of no gene mutation in WT mice. Nevertheless, a widespread distribution of ThioS- and 6E10-positive plaques was evidently observed in the hippocampus of APP/PS1 mice, with good overlap of two images, while TSA treatment significantly reduced both number and area of the Aβ immunoreactive plaques in the hippocampus of APP/PS1 mice (*p* < 0.05) (Fig. 2b, c). Next, we measured soluble neurotoxic Aβ oligomers with Western blot using 6E10 and D54D2 antibodies (Fig. S3A). Similar to immunostaining results, higher levels of 6E10 and D54D2 immunopositive blots around 10 kDa (presumably Aβ oligomers) were found in the hippocampus of vehicle-treated APP/PS1 mice than vehicle-treated WT mice (6E10: *p* < 0.01, D54D2: *p* < 0.001), while the increase in the presumable Aβ oligomer was significantly reduced in TSA-treated APP/PS1 mice (6E10: *p* < 0.05, D54D2: *p* < 0.001) (Fig. S3B and C). Furthermore, by using Simoa assay, we justified that TSA noticeably downregulated the levels of soluble Aβ_40_ (*p* < 0.01) and Aβ_42_ (*p* < 0.05) in the hippocampal homogenates of APP/PS1 mice (Fig. 2d). These data strongly demonstrated that TSA diminished both insoluble Aβ deposition and soluble Aβ in the hippocampus of APP/PS1 mice. Besides, TSA also suppressed microgliosis in the hippocampus of APP/PS1 mice (Fig. S3D-F and see Supplementary Results).

As the level of Aβ in the brain is dependent on the dynamic equilibrium between Aβ production and Aβ clearance, the TSA-induced Aβ reduction might resulted from Aβ production inhibition or Aβ clearance enhancement. However, our further examination showed TSA did not affect Aβ production, because it did not change the levels of full-length APP (flAPP), β-secretase (BACE1), and C-terminal APP fragments (CTF) (*p* > 0.05) (Fig. S4A and C-F). Meanwhile, the levels of IDE and NEP, two major hydrolytic enzymes for degrading extracellular Aβ, also did not change by TSA (*p* > 0.05) (Fig. S4B and G-H), suggesting that TSA did not promote Aβ extracellular degradation. In contrast, intracellular ubiquitin-proteasome pathway, not autophagy, was involved in TSA-induced removal of Aβ in the brain (Fig. S5A-F and see supplementary results).

Since removal of Aβ from blood can reduce the Aβ levels in the brain [23], we also examined whether TSA treatment affected peripheral Aβ clearance in APP/PS1 mice. The results of Simoa assay demonstrated that TSA treatment evidently lowered both Aβ_40_ (*p* < 0.01) and Aβ_42_ levels (*p* < 0.05), as well as their total level (*p* < 0.01) in the plasma of APP/PS1 mice (Fig. 2e), implying that TSA might also promote peripheral clearance of Aβ in the APP/PS1 mice. Since the low-density lipoprotein receptor-related protein 1 (LRP1), as the primary receptor, plays a role in transporting Aβ across the BBB into periphery, we further examined the expression level of LRP1 in the hippocampus. As shown in Fig. S5H, there was no difference in the levels of LRP1 among four groups (*p* > 0.05), indicating that the TSA-induced Aβ removal from brain was not mediated by LRP1.

### TSA, by promoting the combination of albumin with Aβ, enhanced Aβ transport toward the periphery and Aβ endocytosis in the endothelial cell

**Fig. 3:**
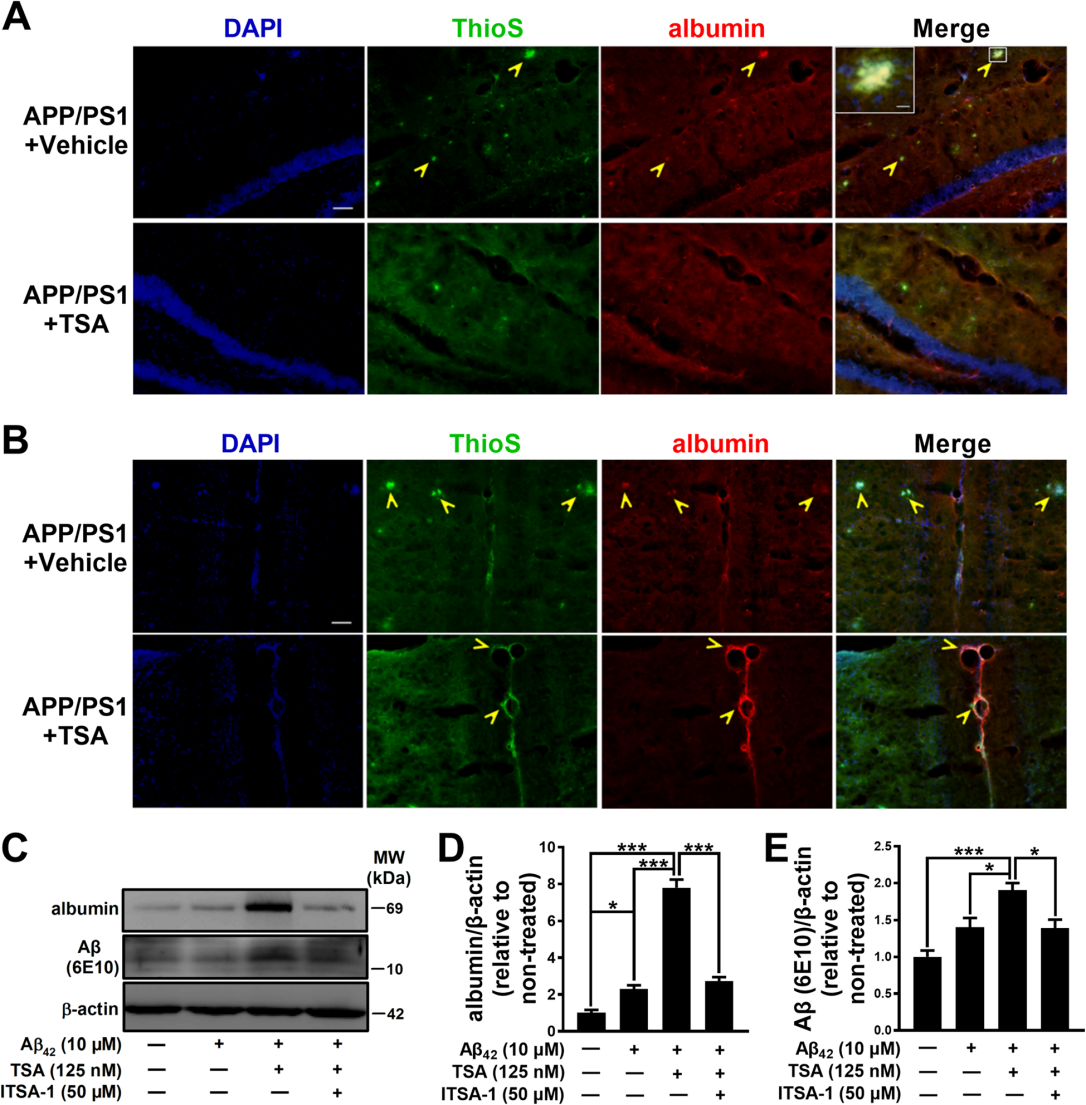
TSA shifted albumin-Aβ complexes to the blood vessels of cerebral falx in APP/PS1 mice and promoted the endocytosis of Aβ oligomers by bEnd.3 cells. **a**, **b** Representative images showing Aβ co-localized with albumin in the hippocampus of APP/PS1 mice. Thioflavin S (ThioS), green; albumin, red; DAPI, blue. Scale bar in **a**: 200 μm. Scale bar in **b**: 10 μm. Yellow arrows indicate representative positive staining. **c** Western blots and **d**, **e** quantitative analysis for albumin and Aβ (6E10) in bEnd.3 cells. *n* = 3–5 per group. *p < 0.05 and ***p < 0.001

Based on the above results, supposing that the clearance and transport of Aβ might be mediated through binding with other proteins, we performed Co-IP and LC-MS/MS to identify the proteins that interacted with Aβ in the hippocampus of APP/PS1 mice. We found that, in the candidates identified by LC-MS/MS, the substance most close to the molecular size in the band of SDS-PAGE gel was albumin (Fig. S6A and see Supplementary Results). So we speculated that the interaction between Aβ and albumin might exist in the hippocampus of APP/PS1 mice. To justify this hypothesis, we further performed an immunofluorescence double-staining experiment and found an obvious co-localization of Aβ plaque and albumin in the hippocampal sections of APP/PS1 mice (Fig. 3a). Moreover, the albumin expression decreased with the reduction of Aβ plaques after TSA treatment. Additionally, the results of Co-IP further confirmed that there was an interaction between albumin and Aβ in the hippocampus of APP/PS1 mice (Fig. S6B). Altogether, these results indicated that albumin might be involved in TSA-induced Aβ clearance in APP/PS1 mice.

In view of albumin as an important transporter in the body [24], we speculated that the central Aβ clearance by TSA is most likely the result of albumin combining Aβ and transporting it outwards. Next, we examined the distribution of albumin-Aβ complexes in the brain by immunofluorescence double staining. As shown in Fig. 3b, albumin-Aβ complexes were indeed scattered in the brain tissues and blood vessels of APP/PS1 mice. Moreover, the complexes around the blood vessels of cerebral falx increased with the decrease of Aβ in the brain tissue after TSA treatment. This result indicated that TSA might promote albumin-mediated the transport of Aβ to periphery through the blood vessels. Therefore, we further examined the level of Aβ-albumin complexes in plasma and found that the level of Aβ-albumin complexes in plasma decreased after TSA treatment in APP/PS1 mice (Fig. S6C), which is consistent with the changes of Aβ and Aβ-albumin complexes in the brain. This finding not only confirmed the interaction between Aβ and albumin, but also suggested a close relationship between the levels of Aβ in the central brain and peripheral plasma after chronic TSA treatment.

To further verify the above results, we then detected the effects of TSA on the levels of albumin and Aβ oligomer in bEnd.3 cells (endothelial cells) by Western blot. As shown in Fig. 3c, d, the expression of albumin in bEnd.3 cells was upregulated in the presence of Aβ_42_ (*p* < 0.05), while that was much higher in co-application of Aβ_42_ and TSA (*p* < 0.001). The high expression of albumin by TSA was reversed by ITSA-1, a specific inhibitor of TSA (*p* < 0.001). Furthermore, the endocytosis of Aβ oligomers by bEnd.3 cells was enhanced more prominently in Aβ_42_ + TSA group than Aβ_42_ alone group (*p* < 0.05), whereas that was markedly attenuated in Aβ_42_ + TSA + ITSA-1 group (*p* < 0.05) (Fig. 3e). These findings suggest that TSA treatment increased albumin level and promoted the endocytosis of Aβ oligomers in bEnd.3 cells.

### TSA dramatically elevated the albumin expression in microglia, inhibited Aβ aggregation, and promoted phagocytosis of Aβ oligomers

**Fig. 4:**
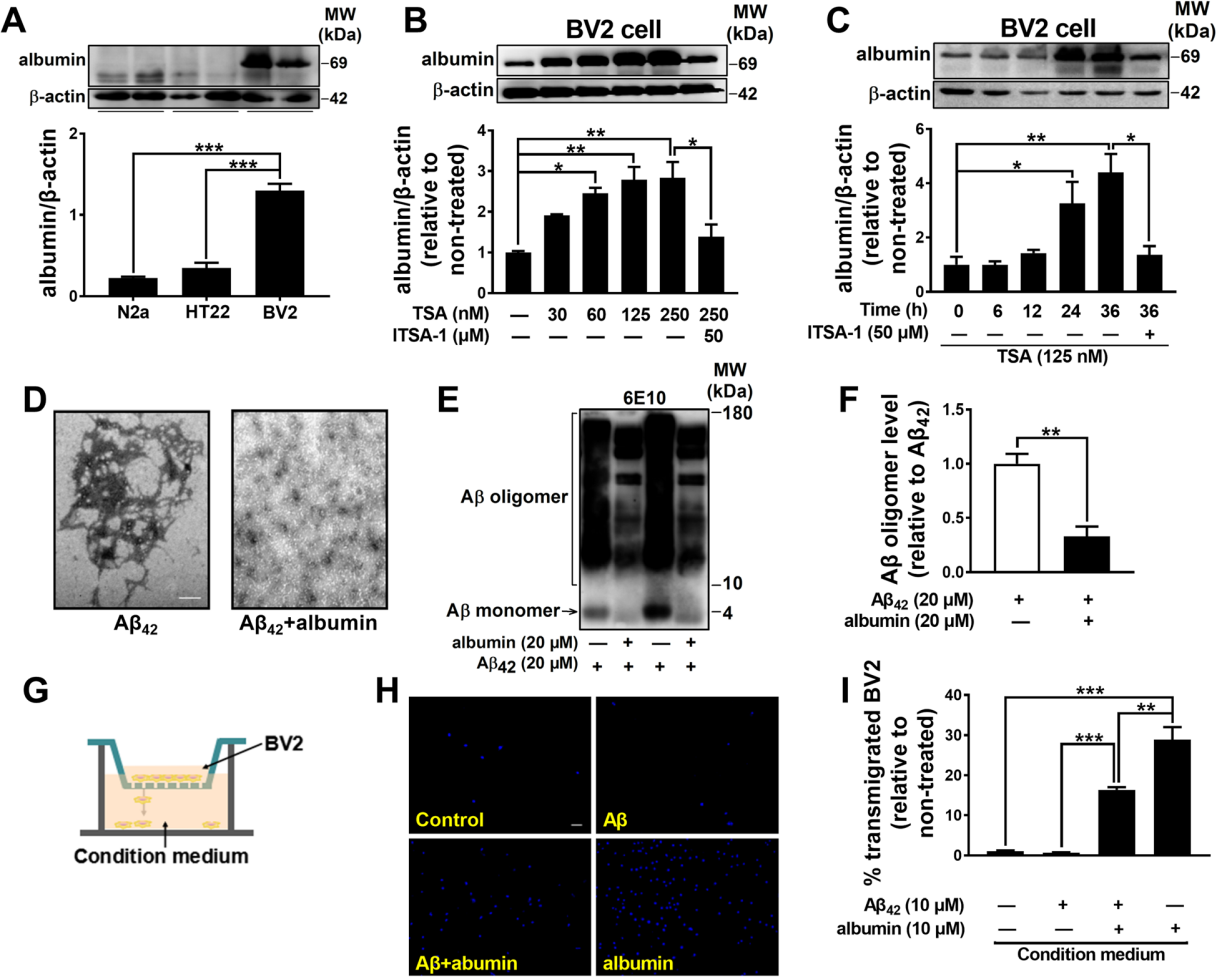
TSA elevated albumin expression in BV2 cells, and albumin inhibited Aβ aggregation and facilitated microglial migration. **a** Western blots and quantitative analysis for albumin in N2a cells, HT22 cells, and BV2 cells. *n* = 3 per group. **b**, **c** Western blots and quantitative analysis for albumin in BV2 cells treated by TSA with different concentrations and different treatment times. *n* = 3 per group. **d** Representative TEM images of Aβ_42_ aggregation in the presence or absence of mouse albumin. Conditions: Aβ_42_, 20 μM; mouse albumin, 20 μM; 24 h incubation; 37 °C; constant agitation. Scale bar, 200 nm. **e** Western blots and **f** quantitative analysis for neurotoxic Aβ oligomers with 6E10 in presence or absence of Albumin. *n* = 4 per group. **g** Schematic diagram of transwell system. BV2 cells were seeded onto 8-μm transwell inserts in 24-well plates in the absence or presence of Aβ_42_ and/or albumin. **h** Representative images of the transmigrated DAPI-labeled BV2 cells in a transwell chamber. Scale bar, 10 μm. **i** Quantification of BV2 cell migration induced by 10 μM Aβ_42_ and/or 10 μM albumin. *n* = 3 per group. *p < 0.05, **p < 0.01, and ***p < 0.001

Microglia, as the primary innate immune cells, have been demonstrated to play a prominent role in Aβ clearance by their phagocytosis in the brain [25], and albumin is also expressed at mRNA and protein levels in human microglia [26]. Here, we further investigated the effects of TSA on the level of albumin and the phagocytosis of Aβ in BV2 cells (microglia cells). Western blot analysis revealed that albumin was mainly expressed in BV2 cells compared to N2a and HT22 cells (Fig. 4a), and the albumin levels in BV2 cells increased with the increase of TSA concentrations (60 nM: *p* < 0.05, 125 nM: *p* < 0.01, 250 nM: *p* < 0.01), with maximal effect observed at 125 nM (Fig. 4b). The levels of albumin in BV2 cells also increased with the increase of TSA (125 nM) treatment time (24 h: *p* < 0.05, 36 h: *p* < 0.01) (Fig. 4c). The dose- and time-dependent increase of albumin expression by TSA can be also reversed by a specific TSA inhibitor ITSA-1 (*p* < 0.05). These data clearly confirmed that TSA greatly enhanced the expression of albumin in BV2 cells.

In light of the above findings, we supposed that mouse albumin might directly affect the aggregation of Aβ. TEM images showed that the incubation of Aβ_42_ with mouse albumin resulted in a dramatic inhibition for the Aβ fibril formation compared with Aβ_42_ alone (Fig. 4d). Western blot analysis showed that mouse albumin apparently reduced neurotoxic Aβ oligomers (*p* < 0.05) (Fig. 4e, f). These findings suggested that mouse albumin could directly bind to Aβ and effectively inhibit Aβ aggregation.

**Fig. 5:**
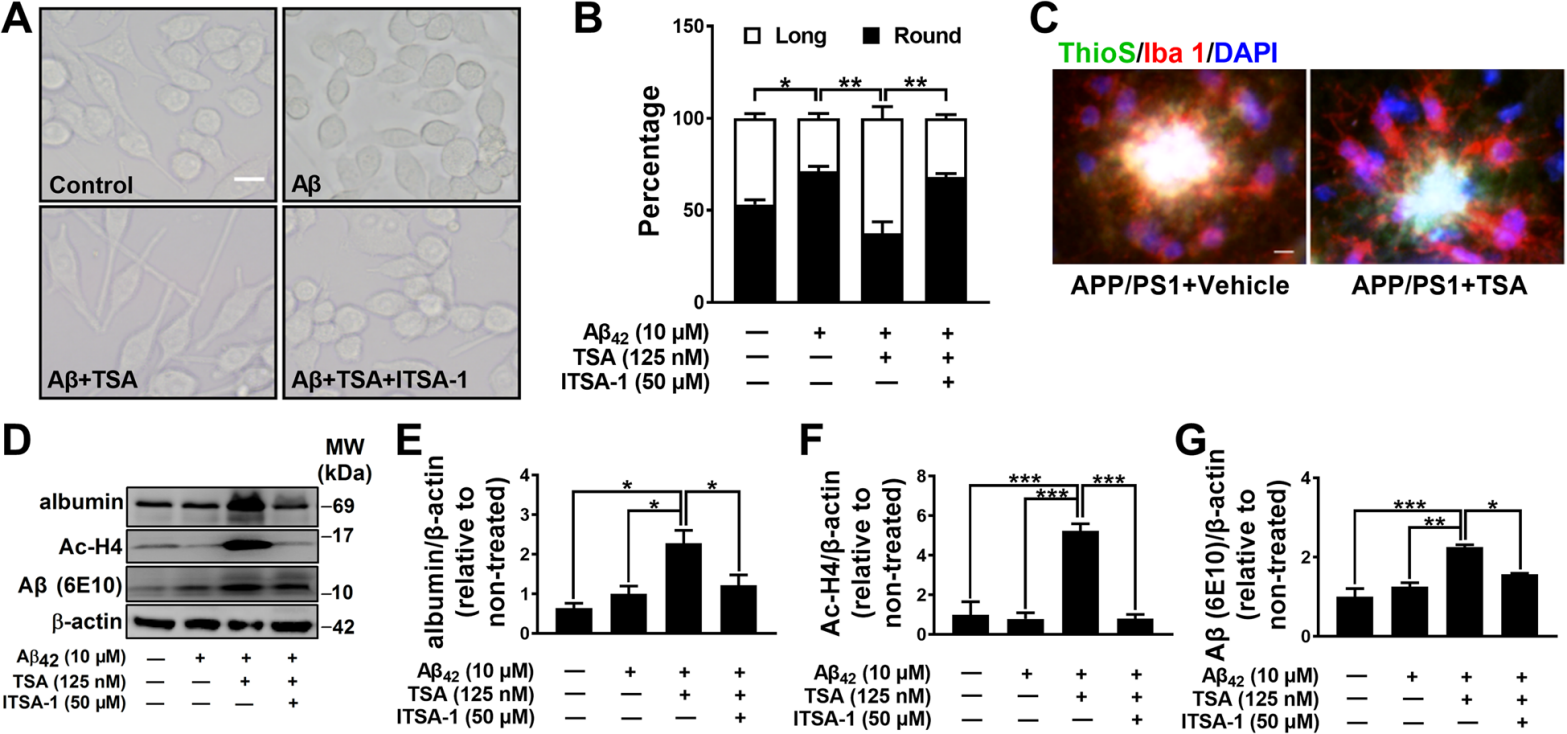
TSA structurally and functionally promoted the phagocytosis of Aβ by BV2 cells. **a** Representative images of BV2 microglia in the different treatment groups. Scale bar, 10 μm. **b** Quantification of each morphological type of BV2 cells in the different treatment groups. *n* = 3 per group. **c** Representative images of microglia in APP/PS1 mice treated with vehicle or TSA. Scale bar, 10 μm. **d** Western blots and **e**–**g** quantitative analysis for albumin, Ac-H4 and Aβ oligomers in BV2 cells. *n* = 3–5 per group. *p < 0.05, **p < 0.01, and ***p < 0.001

The interaction of albumin with Aβ and the cluster of microglia around Aβ plaques in the APP/PS1 mice strongly suggested that albumin might be involved in the migration and phagocytosis of microglia. To validate this hypothesis, the effects of albumin on BV2 cell migration were firstly detected using an in vitro transwell system. As illustrated in Fig. 4g–i, the migration of BV2 cells was slightly inhibited by Aβ (*p* > 0.05), while albumin evidently increased BV2 cell migration (*p* < 0.01) and reversed the inhibitory effects of Aβ (*p* < 0.001), indicating that the combination of albumin and Aβ facilitated microglial migration to Aβ. Further, we observed the morphological changes and the phagocytosis of BV2 cells in the presence of Aβ and TSA. As shown in Fig. 5a, b, normal BV2 cells were mostly round or long with a ratio about 50%, while Aβ-treated BV2 cells displayed more round shape with less and shorter pseudopodia (*p* < 0.05). In the presence of TSA, the pseudopodia of BV2 cells became more and longer even with the same concentration of Aβ_42_ (*p* < 0.01), while this effect of TSA could be inhibited by TSA inhibitor ITSA-1 (*p* < 0.01). In concordance with this, TSA treatment promoted microglia to adopt a ramified shape and increased their pseudopodia in the hippocampus of APP/PS1 mice (Fig. 5c). The increase of pseudopodia indicates the enhancement of phagocytic ability of BV2 cells [27]. To further assess the effects of TSA on Aβ phagocytosis in BV2 cells, we next examined the levels of intracellular Aβ oligomers, albumin, and Ac-H4 by Western blot. As shown in Fig. 5d–g, compared to Aβ_42_ alone treatment, co-application of TSA and Aβ_42_ not only upregulated the levels of albumin (*p* < 0.05) and Ac-H4 (*p* < 0.001), but also significantly elevated Aβ oligomers (*p* < 0.01) in BV2 cells. All of these elevations induced by TSA were also markedly suppressed by ITSA-1 (*p* < 0.05 or *p* < 0.001). These results above indicated that TSA treatment structurally and functionally promoted the phagocytosis of Aβ oligomers by BV2 cells.

## Discussion

The present study confirmed that chronic administration of TSA could ameliorate the short-term and long-term memory of APP/PS1 mice simultaneously in novel object recognition and Morris water maze tests. These behavioral findings further complemented previous experimental researches [15, 28, 29] and supported our results from big data analysis that TSA might be a potential anti-AD drug. As a key feature of AD, Aβ deposition is mainly located in the brain regions involved in cognitive functions, particularly the hippocampus [4], which caused deterioration of the ability of those brain regions to orchestrate cognition. At present, there are several divergent reports on the effects of TSA on Aβ deposition. For example, Yang et al.’s reports showed that administration of TSA in APP/PS1 mice prevented the formation of new Aβ deposition but increased the size of existing plaques [16]. Prasad and colleagues found that TSA was conducive to phagocytose Aβ by astrocytes [30]. Similarly, several studies also found that TSA analogs reduced Aβ deposition [31, 32]. Here, we employed multiple experimental techniques to detect the Aβ in the brain of mice. By combined application of ThioS staining and Aβ immunostaining (6E10), we demonstrated that TSA could significantly reduce the number and area percentage of Aβ plaques in the hippocampus of APP/PS1 mice. Considering that Aβ plaques maybe only reflect the levels of those insoluble aggregated Aβ fibrils, not including soluble neurotoxic Aβ oligomers, we performed immumoblotting with 6E10 and D54D2 antibodies to detect soluble Aβ level of mice and found that TSA effectively decreased the level of hippocampal soluble Aβ, presumably Aβ oligomers, in the APP/PS1 mice. Then, by using ultra-sensitive Simoa assay, our data revealed that TSA treatment decreased both Aβ_40_ and Aβ_42_, two major forms of Aβ, in the hippocampus of APP/PS1 mice. These findings indicated that TSA not only reduced the Aβ plaques but also decreased the levels of presumable Aβ oligomers in the hippocampus of APP/PS1 mice. In addition, the present study also found that TSA treatment significantly alleviated abnormal microglial proliferation in the hippocampus of APP/PS1 mice. Since microglial proliferation has been also recognized as a characteristic of AD in humans and blocking microglial proliferation can improve short-term memory and synaptic density in the hippocampus of APP/PS1 [33], we supposed that the reductions of Aβ level and microglial proliferation in the brain might contribute to the TSA-induced improvement of cognitive behaviors in the APP/PS1 mice. These pathological findings established a solid foundation for the follow-up molecular researches.

The imbalance between Aβ production and Aβ clearance results in Aβ accumulation and aggregation in the brain [34]. So, the potential mechanisms for TSA decreasing Aβ accumulation in the brain might refer to the inhibition of excess Aβ production and/or the promotion of Aβ degradation and clearance. To clarify the principles of TSA in decreasing Aβ, we examined the expression levels of the proteins associated with amyloidogenic processing, Aβ degradation, and Aβ transport. Our results demonstrated that TSA did not affect the levels of flAPP, CTF, and BACE1 in the hippocampus of APP/PS1 mice, which is consistent with Yang et al.’ observations [17], suggesting that TSA did not target Aβ-producing activity but rather Aβ clearance. It is known that the clearance of Aβ from the brain is involved in several mechanisms, including enzymatic degradation, uptake by microglial or astrocytic phagocytosis, proteasome degradation, and peripheral clearance. The first, extracellular Aβ can be rapidly degraded by several enzymes, such as NEP and IDE. However, there was no significant difference in the levels of IDE and NEP in mouse hippocampus among groups, suggesting that TSA did not affect the enzymatic degradation pathway of Aβ. Secondly, Aβ is also degraded by two major intracellular pathways, UPP and ALP. Although previous studies found that TSA was able to induce autophagy [18, 35, 36], we did not find any significant change in the levels of autophagy-related proteins in the hippocampus of mice by TSA. Instead, we found that TSA slightly increased the level of monomeric ubiquitin in the hippocampus of WT mice, which shared a number of similarities with Tian et al.’s [37] findings that chronic TSA treatment increased monomeric ubiquitin level, including ubiquitin B and ubiquitin A. We also justified that APP/PS1 mice had a much higher level of ubiquitin monomer relative to WT mice, which was in line with previous results by Tseng and colleagues [38] in which proteasomal dysfunction resulted in the increases of Aβ accumulation and monomeric ubiquitin in AD mice brains. We noticed that TSA treatment significantly decreased the level of ubiquitin monomer in the hippocampus of APP/PS1 mice, suggesting that TSA might be through UPP to reduce Aβ level, thereby decreasing the level of ubiquitin monomer. In addition, according to the “peripheral Aβ sink” hypothesis, Aβ between the brain and periphery is in equilibrium. Reduction of peripheral Aβ would help with the efflux of central Aβ in the brain or cerebrospinal fluid (CSF) [39–41]. Our results by Simoa assay disclosed that chronic TSA treatment significantly decreased the levels of Aβ_40_ and Aβ_42_ in plasma of APP/PS1 mice. So, we speculated that the peripheral clearance is at least partially involved in the reduction of central Aβ induced by TSA. But what mediated the transport of Aβ from brain to periphery?

In the present study, we firstly examined the expression level of LRP1, a main cell surface transporter protein participated in both Aβ endocytosis and transcytosis across the BBB [42–44]*.* But no significant change in LRP1 level was found among groups. In light of that Aβ transport depends on the interaction between Aβ and variety proteins [45, 46], interestingly, our Co-IP and LC-MS/MS analysis indicated that there was a close interaction between albumin and Aβ in the hippocampus of APP/PS1 mice. Furthermore, we found that hippocampal albumin-Aβ complexes decreased with the decrease of Aβ after TSA treatment in APP/PS1 mice. This may be related to the interaction of proteins and the transportation of albumin. Albumin is mainly synthesized in the liver, with high ligand binding and transport capacity [24, 47–50]. Hence, we speculated that albumin, as an important protein carrier, might be involved in the transport of Aβ during the treatment with TSA. To verify this hypothesis, we performed double immunofluorescence staining and demonstrated that a large number of albumin-Aβ complexes were shifted to and clustered in the blood vessels around cerebral falx in APP/PS1 mice after TSA treatment, implying that TSA-induced Aβ clearance is most likely mediated by the combination and transportation of albumin. So, the levels of albumin and Aβ in the hippocampus should be dynamic, depending on the TSA treatment. Indeed, we found that the expression levels of albumin and Aβ decreased in the hippocampus of APP/PS1 mice while both of them increased around brain blood vessels after chronic treatment with TSA. Considering the dynamic changes of Aβ-albumin complexes with clearance and transfer of Aβ after long-term TSA treatment in vivo, we proposed that the early elevated Aβ-albumin complexes in hippocampus might be transported to the periphery. Hence, the expression level of albumin bound to Aβ decreased with the clearance of Aβ in the hippocampus. In addition, our studies revealed that treatment of bEnd.3 cells with TSA significantly upregulated albumin level and enhanced the endocytosis of Aβ, further supporting the hypothesis that the TSA-induced high expression of albumin in endothelial cells may be beneficial to combining and transporting Aβ across cerebral blood vessels toward the periphery in APP/PS1 mice. That is also why albumin-Aβ complexes were vastly concentrated around cerebral blood vessels in TSA-treated APP/PS1 mice.

Albumin is not only a high-abundance protein in plasma, but also a major component of most extracellular fluids including CSF, interstitial fluid (ISF) and lymph [51–54]. It has multifunctional properties including transportation of hormones, fatty acids, drugs, and metabolites, protective action on neuronal and glia cells, antioxidant activity, and anti-inflammatory activity [55, 56]. Owing to its multi-functionality, albumin has been implicated in many disease conditions of the brain, including AD. Extensive evidences have shown that albumin has a high affinity for Aβ, which can bind to and transport around 90% circulating Aβ. It is worth noting that a lower serum albumin concentration has been reported in AD patients, compared to those healthy counterparts [57, 58]. Several clinical studies also showed that plasma exchange with human albumin or intravenous administration of albumin ameliorated cerebral pathology, memory behavior, and language functions in AD patients [58–60]. These facts correlate well the decreased plasma albumin with the accumulated central Aβ, being consistent with “peripheral Aβ sink” hypothesis and suggesting that albumin might be a new target for the clearance of Aβ. Interestingly, Vanhaecke reported that TSA induced a higher albumin secretion in cultured rat hepatocytes from 7th day after TSA treatment [61]. Accordingly, we speculate that the peripheral effects of TSA might also play an important role in the central Aβ clearance. The chronic treatment with TSA in the present in vivo study might elevate the plasma albumin level in AD mice, which could be beneficial for Aβ binding, transportation, and clearance in the periphery. Indeed, our further experiments demonstrated that the levels of both Aβ and Aβ-albumin complexes in plasma were all declined by chronic TSA treatment in APP/PS1 mice. Of course, the detailed mechanisms involved in plasma Aβ clearance by TSA are still to be investigated.

Microglia, the primary innate immune effector cells in the CNS, rapidly activate upon encountering tissue damage or injury and transform into active phagocytic microglia with high capacity for phagocytic removal of cellular debris or damaged neurons [62, 63]. It has been reported that microglial phagocytosis of Aβ was impaired in the brains of AD patients and AD mouse models [64]. Therefore, the enhancement of microglial phagocytic activity to clear Aβ deposits plays an important role in AD progression. Ahn et al. found that microglia could synthesize albumin in the human brain [26], but its function and regulation mechanism remain unclear. Based on these issues, by using Western blot and different cell lines including N2a mouse neuroblastoma cells, HT22 murine hippocampal neuronal cells and BV2 murine microglial cells, we compared the expression levels of albumin in these cell lines. As expected, albumin was mainly expressed in BV2 cells, and TSA treatment enhanced albumin expression of BV2 cells in a dose- and time-dependent manner, while these effects could be repressed by ITSA-1. Moreover, albumin can also prevent Aβ polymerization [47]. As mentioned by Choi [21] and Picón-Pagès [65], human serum albumin suppressed Aβ aggregation by binding to the oligomeric or polymeric Aβ and blocking a further addition of peptide and attenuated Aβ neurotoxicity. We also confirmed that mouse albumin directly bound to Aβ and efficiently suppressed the formation of Aβ fibrils, suggesting that the antagonistic effects of TSA on Aβ-induced neurotoxicity was mediated by elevating albumin expression in mouse brain microglia, which then bound to Aβ and prevented its aggregation. Considering the fact that microglia always surround Aβ plaques, we wondered that whether the microglia expressed albumin induced microglia migration to Aβ. The transwell migration assay clearly indicated that albumin, rather than Aβ, indeed promoted the migration of BV2 cells. These findings indicated that Aβ plaque itself did not induce the migration and aggregation of microglia, the microglial migration and aggregation might be mediated by some protein binding to Aβ, such as albumin. The combination of albumin and Aβ might be important and necessary in the microglial migration to Aβ and the microglial phagocytosis of Aβ. It is possible that albumin acts as a signal molecule in Aβ recognition and mediates the phagocytosis and clearance of Aβ by microglia. Indeed, our in vitro Western blot with cultured microglia showed that TSA treatment not only upregulated the levels of albumin and acetylated histone H4, which is in accordance with Takuma et al.’s findings that the acetylation of histone H3K14 was increased by TSA in PC12 cell [66], but also increased the microglia pseudopodia and enhanced the phagocytosis of Aβ in APP/PS1 mice.

### Limitations for this study

Our current study has some limitations. Although our study showed that TSA, as a HDAC inhibitor, could promote Aβ clearance by elevating albumin expression in microglia and microvascular endothelial cells, the precise signaling pathways that are involved in the upregulation of albumin by TSA remain to be elucidated. Additionally, cognitive impairments and AD-related pathology were improved in an AD animal model through chronic TSA treatment. However, the therapeutic benefit of this in patients with AD remains unknown. Further clinical trials are needed to confirm our results.

## Conclusions

In summary, this study further confirmed that TSA could effectively improve the cognitive behaviors and AD-like pathology in APP/PS1 mice. We systematically explore the effects of TSA on Aβ clearance pathways and find that the mechanisms underlying the protective roles of TSA are mainly involved in the enhancement of phagocytosis of Aβ by microglia and the promotion of endocytosis of Aβ by microvascular endothelial cells. Moreover, the upregulation of albumin by TSA plays important mediating roles in the Aβ clearance including promoting Aβ transport, preventing Aβ aggregation, and inducing microglia migration to Aβ (Fig. S7). The study uncovered a new mechanism for TSA in modulation of Aβ clearance and suggested that TSA would be an ideal candidate for further clinical tests as a therapeutic medicine of AD.

## Supplementary Information

**Additional file 1.** Supplementary Information accompanies this paper on the Alzheimer’s Research & Therapy website (https://alzres.biomedcentral.com).

## Data Availability

All data generated in this study are available from the corresponding author on reasonable request.

## Abbreviations

AD: Alzheimer’s disease
Aβ: Amyloid β
TSA: Trichostatin A
HDAC: Histone deacetylase
LTP: Long-term potentiation
NOI: Novel object recognition index
Simoa: Single molecule array
Co-IP: Co-immunoprecipitation
LC-MS/MS: Liquid chromatography-tandem mass spectrometry
APP: Amyloid precursor protein
flAPP: Full-length amyloid precursor protein
CTF: C-terminal fragment
BACE1: β-site APP cleaving enzyme 1
UPP: Ubiquitin-proteasome pathway
ALP: Autophagy-lysosome pathway
NEP: Neprilysin
IDE: Insulin-degrading enzyme
LRP1: Low-density lipoprotein receptor-related protein 1
Ac-H4: Acetylated histone H4
ISF: Interstitial fluid
CSF: Cerebrospinal fluid
BBB: Blood brain barrier
