# Supplementary material for: Trichostatin A ameliorates Alzheimer’s disease-related pathology and cognitive deficits by increasing albumin expression and Aβ clearance in APP/PS1 mice
Source: Alzheimers Res Ther. 2021 Jan 4;13:7. doi: 10.1186/s13195-020-00746-8 (PMC7784383; doi:10.1186/s13195-020-00746-8)
Supplement: Supplementary file 1 — Additional file 1. Supplementary Information accompanies this paper on the Alzheimer’s Research & Therapy website (https://alzres.biomedcentral.com). [file 13195_2020_746_MOESM1_ESM.doc]

**SUPPLEMENTARY INFORMATION**

**Trichostatin A ameliorates Alzheimer’s disease-related pathology and cognitive deficits by increasing albumin expression and Aβ clearance in APP/PS1 mice**

Qiang Su^1†^,Tian Li^1†^, Pei-Feng He^2*^, Xue-Chun Lu^3*^, Qi Yu^2^, Qi-Chao Gao^1^, Zhao-Jun Wang^1^, Mei-Na Wu^1^, Dan Yang^1^, Jin-Shun Qi^1*^

^1^ Department of Physiology, Key Laboratory of Cellular Physiology, Ministry of Education, Shanxi Medical University, Taiyuan, Shanxi 030001, China.

^2^ Institute of Medical Data Sciences and School of Management, Shanxi Medical University, Taiyuan, Shanxi 030001, China.

^3^ Department of Hematology, the Second Medical Center & National Clinical Research Center for Geriatric Diseases, Chinese PLA General Hospital, Beijing, 100853, China.

^†^ With the same contribution

* Corresponding author：

Jin-Shun Qi PhD & Prof.

Department of Physiology,

Shanxi Medical University,

Taiyuan, Shanxi 030001, China,

Tel: +86-351-413-5091
E-mail address: jinshunqi2009@163.com

**Supplementary Methods**

**4 Supplementary Results**

**Supplementary References**

**7 Supplementary Figure Legends**

**7 Supplementary Figures**

**Supplementary Methods**

**Co-Immunoprecipitation (Co-IP) and LC-MS/MS analysis**

In order to remove nonspecifically bound proteins, the hippocampal protein was pre-incubated with normal IgG and protein A/G agarose for 2 h at 4 °C, and then centrifuged at 3000 × g for 5 min. The supernatants were collected and incubated with 6E10 antibody at 4 °C overnight, followed by incubation with protein A/G agarose for 2 h at 4 °C. The resulting immunoprecipitates were resuspended with 1× SDS-PAGE loading buffer and detected by WB as described above. For LC-MS/MS analysis, the gel was visualized by coomassie brilliant blue staining solution (P0017F, Beyotime) after SDS-PAGE electrophoresis and the target gel pieces were cut out, destained and dried as previously described , and then analyzed in MS analysis room of Shanghai Bioprofile biology Co. Ltd (China).

**Supplementary Results**

**TSA suppressed microgliosis in the hippocampus of APP/PS1 mice**

Microglia are considered as the resident immune cells of the brain, reflecting the inflammatory response. The abnormal accumulation of Aβ could induce excessive activation of microglia in the brain, which results in neuronal injury in AD . So, we then detected Iba-1-positive microglia in the hippocampus by immunofluorescence staining. As shown in Fig. S3D, microglia clustered around Aβ plaques in the hippocampus of APP/PS1 mice. Meanwhile, the number and area percentage of microglia in the hippocampus were apparently increased in APP/PS1 + Vehicle group relative to those in WT + Vehicle group (p < 0.001), whereas that was remarkably reduced in APP/PS1 + TSA group (p < 0.01) (Fig. S3E and F). These results suggested that TSA inhibited abnormal microglial proliferation in APP/PS1 mice.

**TSA did not affect Aβ production and Aβ enzymatic degradation**

Amyloid precursor protein (APP) is sequentially cleaved by β-secretase (BACE1) and γ-secretase complex to yield the C-terminal APP fragments (CTF) and Aβ . Since BACE1 is a rate-limiting enzyme for the initial cleavage of APP leading to Aβ production in the amyloidogenic pathway , we firstly detected whether TSA affected the amyloidogenic process of APP through BACE1 regulation. Our results showed that the level of full length APP (flAPP) was notably elevated in APP/PS1 mice than that in WT mice (p < 0.001), while TSA had no effect on the level of flAPP (p > 0.05) (Fig. S4C). Furthermore, Western blot results in all mice indicated that TSA failed to regulate the expression of BACE1 or CTF (p > 0.05) (Fig. S4D and E). However, APP/PS1 mice treated with TSA showed a significant decrease in the level of Aβ and the ratio of Aβ/flAPP compared with vehicle treated APP/PS1 (p < 0.05) (Fig. S4F). Together with the results that TSA did not affect the levels of flAPP, BACE1 and CTF, the study indicated that TSA did not target at Aβ-producing but promoted Aβ clearance. In addition, IDE and NEP are the major hydrolytic enzyme in degrading extracellular Aβ . Using Western blot, we further found that the levels of IDE and NEP were also not changed by TSA (p > 0.05) (Fig. S4G and H), suggesting that TSA did not promote Aβ degradation by NEP and IDE in the hippocampus of APP/PS1 mice.

**Ubiquitin-proteasome pathway, but not autophagy, was involved in TSA-induced removal of Aβ in the brain**

The UPP and ALP are the main intracellular proteolytic pathways for Aβ in the brain . Thus, we examined the effects of TSA on the levels of autophagy-relevant proteins and ubiquitin by Western blot (Fig. S5A and D). The results showed that there was no significant difference in the protein levels of Beclin 1, p62 and the ratio of LC3B-II/LC3B-I in the hippocampus among four groups (p > 0.05), although with a bit increase of p62 level in APP/PS1 + TSA group compared to APP/PS1 + Vehicle group (Fig. S5B-C and E). By contrast, compared to WT + Vehicle group, there was a remarkable increase in the level of monomeric ubiquitin in APP/PS1 + Vehicle group (p < 0.01), while that was significantly decreased in APP/PS1 + TSA group (p < 0.05) (Fig. S5F). These data altogether suggested that UPP, not autophagy, was involved in TSA-induced Aβ clearance.

**Aβ interacted with albumin in the hippocampus of APP/PS1 mice**

In the experiments, Aβ-binding proteins were co-immunoprecipitated from the hippocampal homogenates of APP/PS1 mice with anti-Aβ antibody (6E10), followed by SDS-PAGE electrophoresis and coomassie brilliant blue staining. Afterwards, two prominent bands (T1 and T2) between 55 and 72 kDa were seen on the gel. As shown in the Fig. S6A, the intensity of T2 band (APP/PS1 + TSA group) was weaker than T1 band (APP/PS1 + Vehicle group), which was consistent with the fact that Aβ level was reduced in TSA-treated APP/PS1 mice in comparison to vehicle-treated APP/PS1 mice. These bands were then detected by LC-MS/MS and subjected to data processing and screening with score and intensity. Of the candidates inferred from the peptides identified by LC-MS/MS, we noted that the substance most close to the molecular size displayed in the band of SDS-PAGE gel was albumin, which has been reported to be a binding protein for Aβ . Furthermore, the results of Co-IP showed that in input fractions (positive control), Aβ expressed in all groups even though with different levels. However, in IP fractions, there was no expression of albumin in the co-immunoprecipitated proteins with 6E10 antibody in IgG group (negative control), while that was expressed in WT and APP/PS1 mice. These results validated that there was an interaction between albumin and Aβ in the hippocampus of APP/PS1 mice (Fig. S6B).

**Supplementary References**

1. Matz A, Halamoda-Kenzaoui B, Hamelin R, Mosser S, Alattia JR, Dimitrov M, et al. Identification of new Presenilin-1 phosphosites: implication for gamma-secretase activity and Aβ production. J Neurochem. 2015;133(3):409-21.

2. Heneka MT, Carson MJ, Khoury JE, Landreth GE, Brosseron F, Feinstein DL, et al. Neuroinflammation in Alzheimer's disease. The Lancet Neurology. 2015;14(4):388-405.

3. Selkoe DJ. Alzheimer's disease: genes, proteins, and therapy. Physiol Rev. 2001;81(2):741-66.

4. Ye X, Cai Q. Snapin-mediated BACE1 retrograde transport is essential for its degradation in lysosomes and regulation of APP processing in neurons. Cell Rep. 2014;6(1):24-31.

5. Shirotani K, Tsubuki S, Iwata N, Takaki Y, Harigaya W, Maruyama K, et al. Neprilysin degrades both amyloid beta peptides 1-40 and 1-42 most rapidly and efficiently among thiorphan- and phosphoramidon-sensitive endopeptidases. J Biol Chem. 2001;276(24):21895-901.

6. Vekrellis K, Ye Z, Qiu WQ, Walsh D, Hartley D, Chesneau V, et al. Neurons regulate extracellular levels of amyloid beta-protein via proteolysis by insulin-degrading enzyme. J Neurosci. 2000;20(5):1657-65.

7. Finkbeiner S. The autophagy lysosomal pathway and neurodegeneration. Cold Spring Harb Perspect Biol. 2020;12(3):a033993.

8. Morawe T, Hiebel C, Kern A, Behl C. Protein homeostasis, aging and Alzheimer's disease. Mol Neurobiol. 2012;46(1):41-54.

9. Choi TS, Lee HJ, Han JY, Lim MH, Kim HI. Molecular insights into human serum albumin as a receptor of amyloid-β in the extracellular region. J Am Chem Soc. 2017;139(43):15437-45.

**Supplementary Figure Legends**

**Supplementary Fig. 1.** Experimental design for the animal study. 8-mon-old APP/PS1 mice were treated with TSA (2 mg/kg, i.p.) or vehicle (i.p.) once daily for 30 days before behavioral tests, which was kept during all behavioral tests, including novel object recognition test (NORT) and Morris water maze test (MWMT). To avoid behavioral irritation affecting follow-up experimental results, the mice were sacrificed after two-week recovery from behavioral tests.

**Supplementary Fig. 2.** The visual and motor ability of mice were not influenced by genotype and drug in visible platform test of Morris water maze. (A) The escape latency and (B) the swimming speed of mice in visible platform test. n = 9-10 in each group.

**Supplementary Fig. 3.** TSA treatment decreased the level of Aβ oligomers and suppressed microglial proliferation in hippocampus of APP/PS1 mice. (A) Western blot analysis and (B, C) quantification of Aβ oligomers with 6E10 and D54D2 antibodies in hippocampal homogenates. n = 5 per each group. (D) Representative images showing the proliferated microglia in APP/PS1 mice were suppressed by TSA treatment. Note that Aβ plaques were closely surrounded by microglia in the merged images. Thioflavin-S (ThioS), green; Iba 1, red; DAPI, blue. Scale bar, 200μm; insets: scale bar, 10 µm. (E) The number of Iba 1-positive microglia in the hippocampus (Hip). (F) The percent area of Iba 1-positive microglia in the hippocampus (Hip). n = 6 per each group. *p < 0.05, **p < 0.01 and ***p < 0.001.

**Supplementary Fig. 4.** TSA did not affect the production and proteolytic degradation of Aβ. (A) Western blot analysis and (C, D, E, F) quantification of flAPP, BACE1, CTF and Aβ (D54D2)/flAPP in hippocampus homogenates. (B) Western blots and (G, H) quantitative analysis for IDE and NEP in hippocampus homogenates. n = 5 per each group. *p < 0.05, **p < 0.01 and ***p < 0.001.

**Supplementary Fig. 5.** TSA down-regulated the hippocampal monomeric ubiquitin level in APP/PS1 mice without affecting the levels of autophagy-related protein in the hippocampus of mice. (A) Western blots and (B, C, E) quantitative analysis for p62, Beclin 1 and LC3B-II/LC3B-I in hippocampus homogenates. (D) Western blot analysis and (F) quantification of monomeric ubiquitin in hippocampal homogenates. (G) Western blot analysis and (H) quantification of LRP1 in hippocampal homogenates. n = 5 per each group. *p < 0.05 and **p < 0.01.

**Supplementary Fig. 6.** Identification of the interaction between albumin and Aβ in the hippocampus of APP/PS1 mice. (A) A flow diagram showing the proteomic isolation of Aβ binding proteins. Hippocampal homogenates of mice were co-immunoprecipitated with anti-Aβ antibody (6E10) and resolved by SDS-PAGE, followed by coomassie brilliant blue staining and LC/MS/MS analysis. The first 20 proteins were screened out according to scores and densities, in which albumin was the protein closest to the band in SDS-PAGE gel in molecular weight. (B) Co-immunoprecipitation analysis of Aβ and albumin in hippocampal homogenates from APP/PS1 mice and WT mice. The hippocampal lysate was directly analyzed (Input) or co-immunoprecipitated with an anti-Aβ antibody (6E10) and further blotted with an antibody against albumin (Co-IP). An IgG antibody was used as a negative control. Input, 10% of the total lysate. (C) Plot showing the co-immunoprecipitation of Aβ and albumin in plasma from APP/PS1 mice. The level of plasma Aβ-albumin complexes was decreased in TSA-treated APP/PS1 mice compared to vehicle-treated APP/PS1 mice.

**Supplementary Fig. 7.** A graphical representation of the possible cellular and molecular mechanisms of TSA inhibiting Aβ accumulation and promoting Aβ clearance. Albumin can bind to Aβ and inhibit Aβ aggregation (1). TSA up-regulates the expression and secretion of albumin in microglia and endothelial cells (2). TSA not only enhances the phagocytosis and clearance of Aβ by microglia (3), but also promote the endocytosis and transport of Aβ by endothelial cells (4).

**Supplementary Figures**

**Figure S1**

**
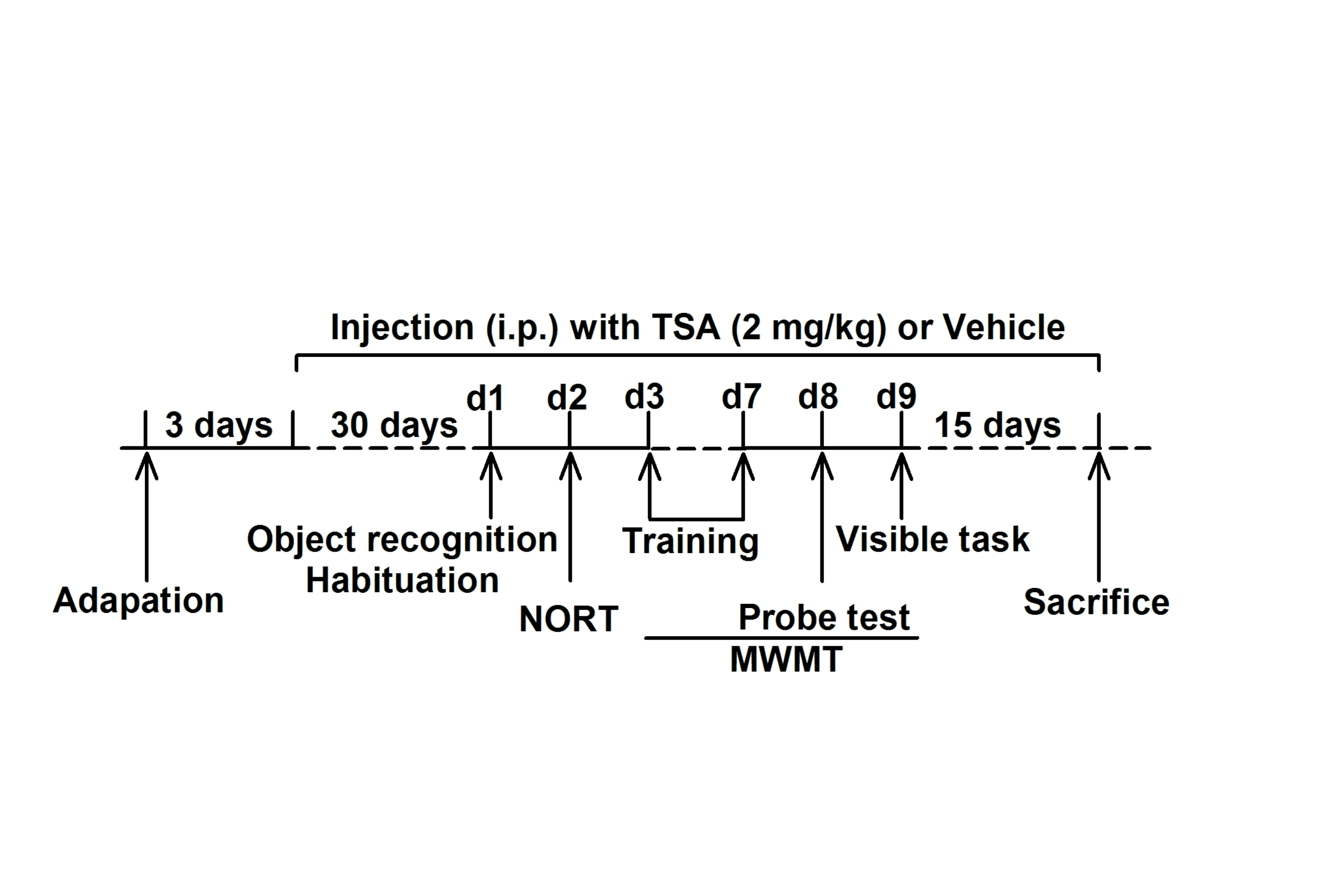
**

**Figure S2**

**
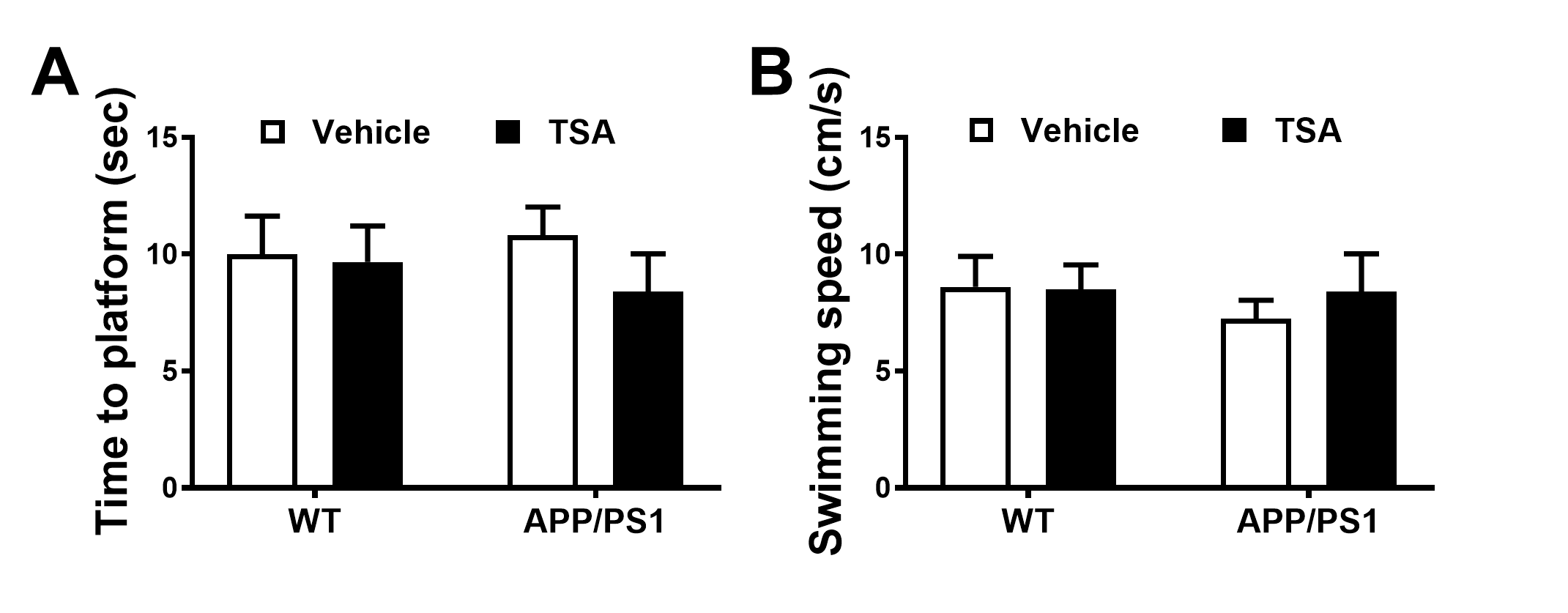
**

**Figure S3**

**
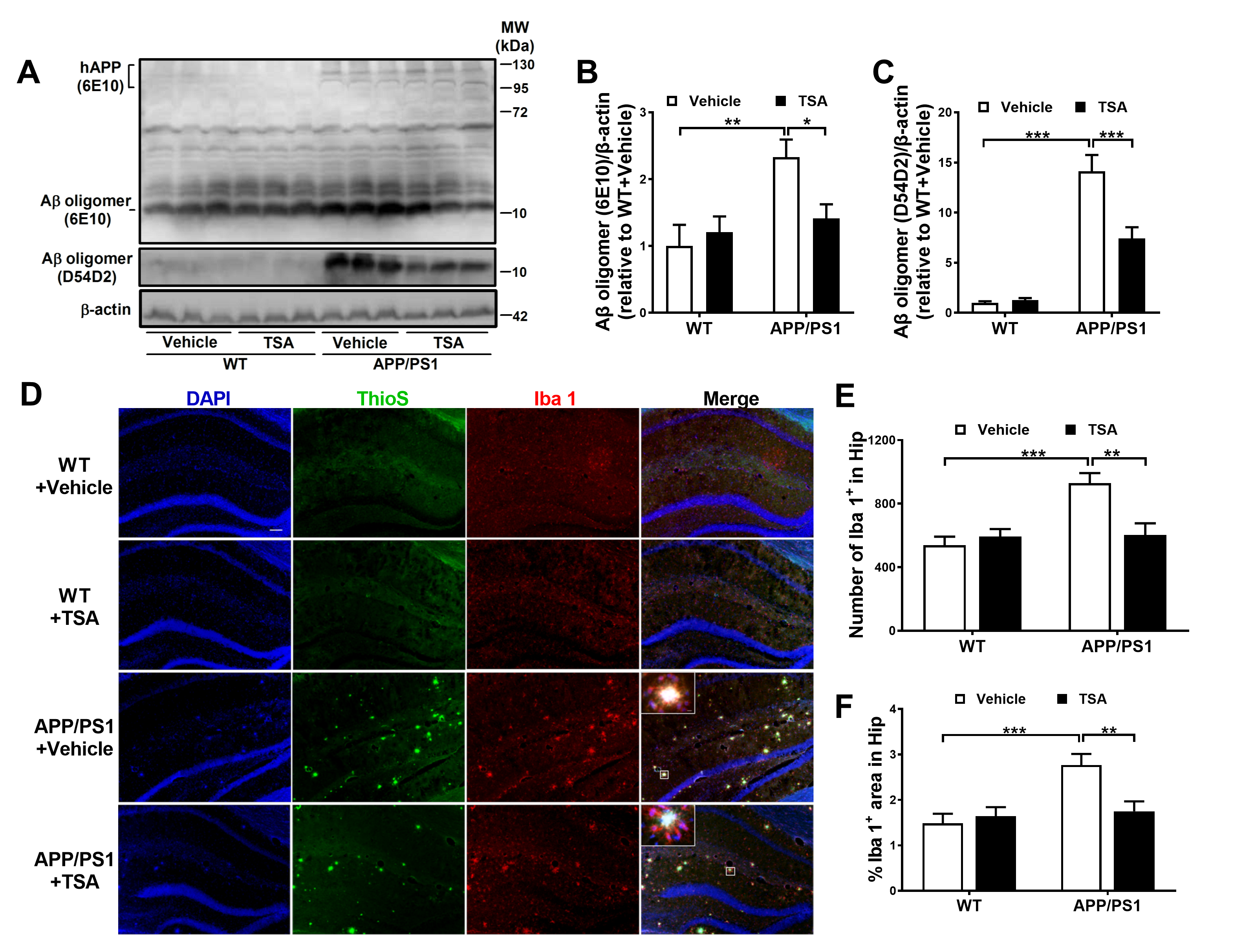
**

**Figure S4**

**
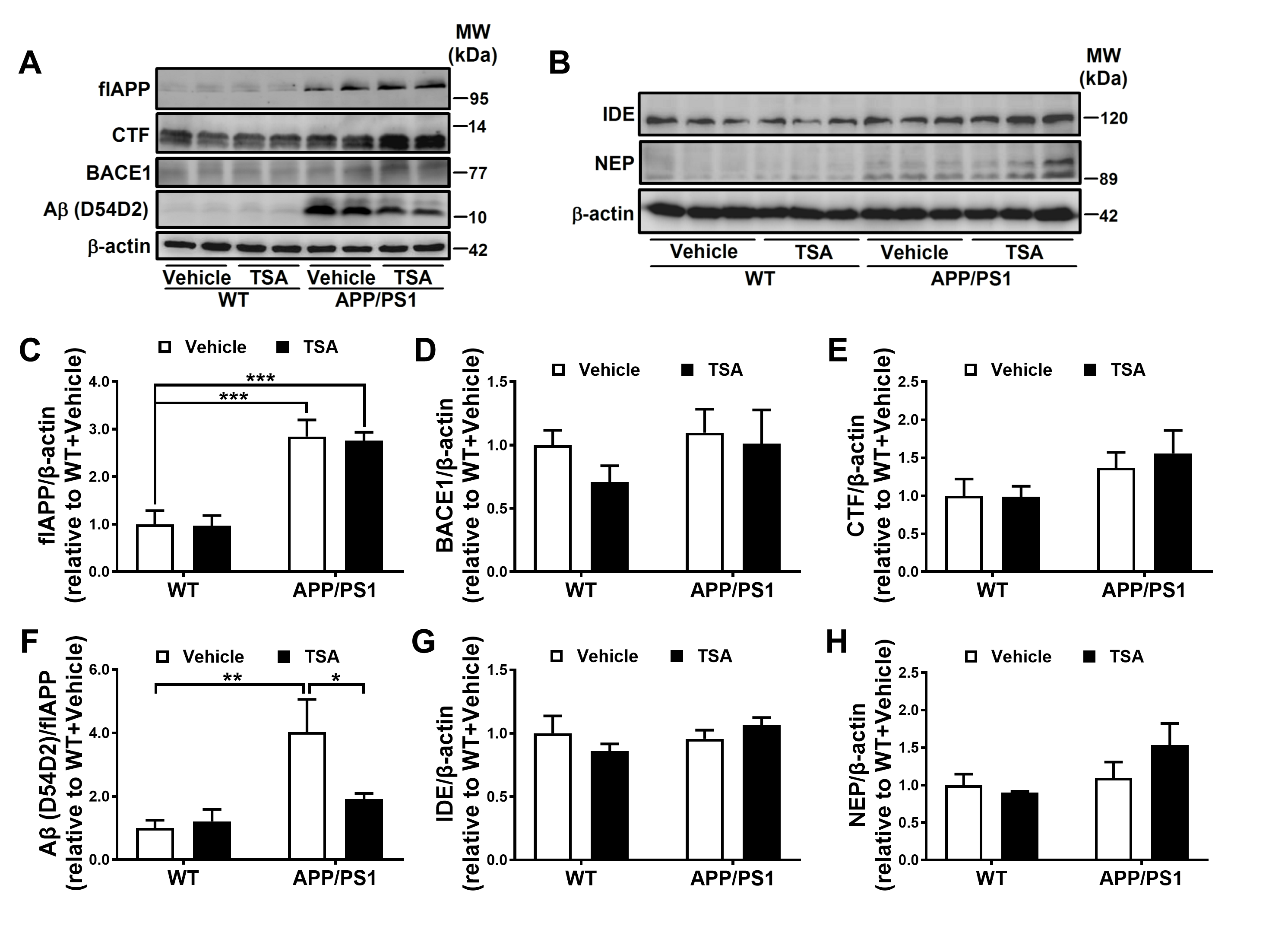
**

**Figure S5**

**
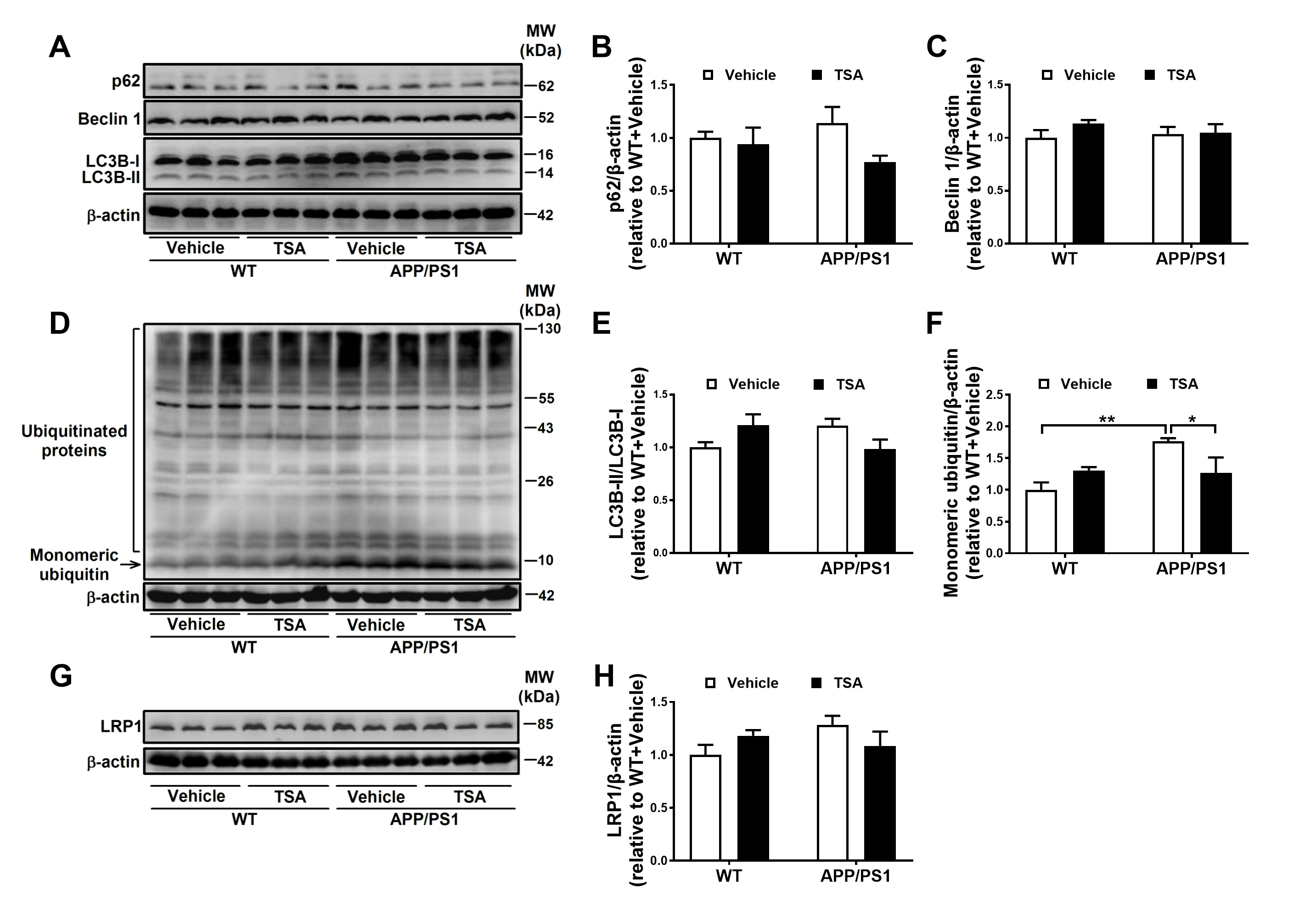
**

**Figure S6**

**
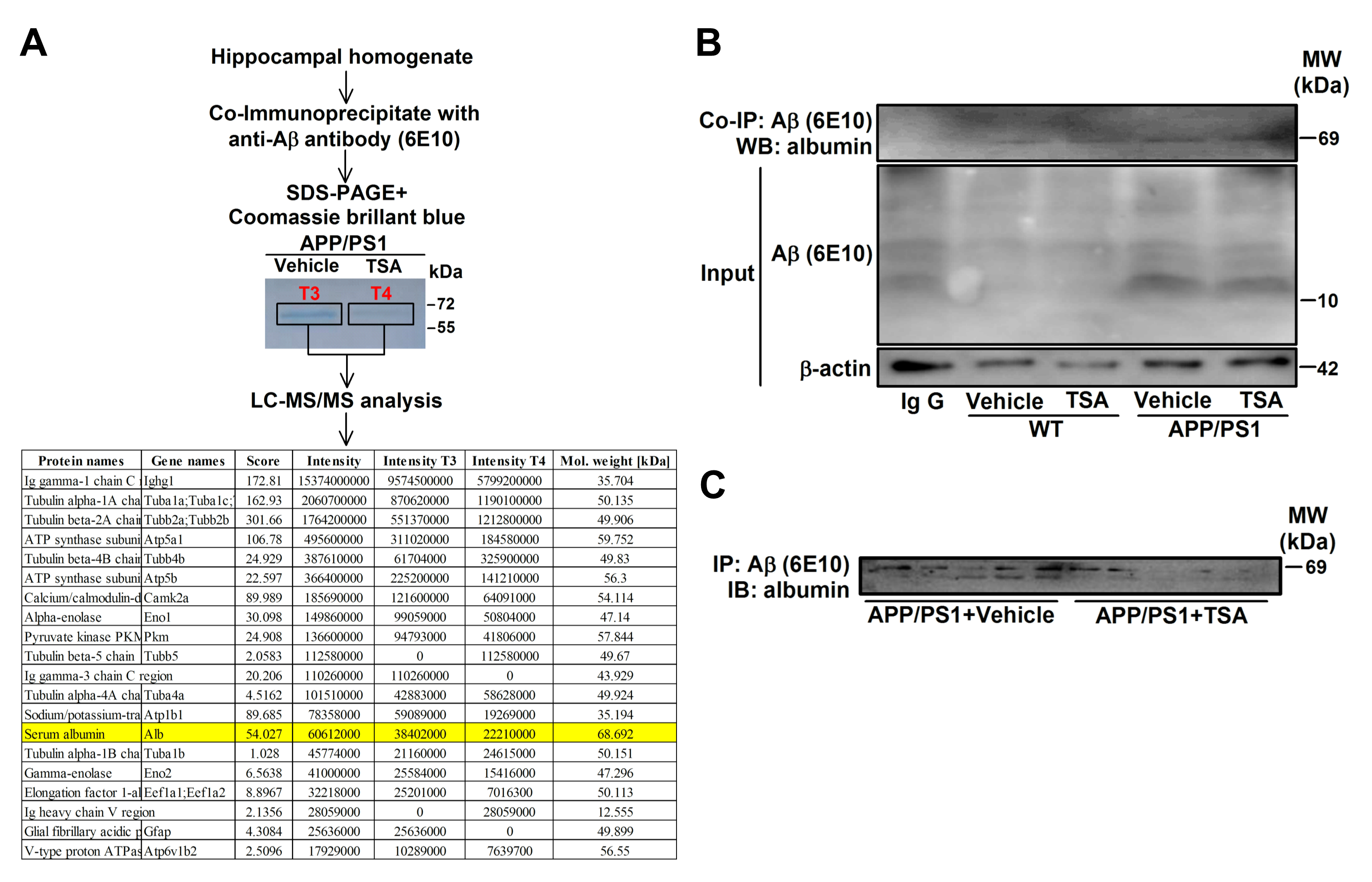
**

**Figure S7**

**
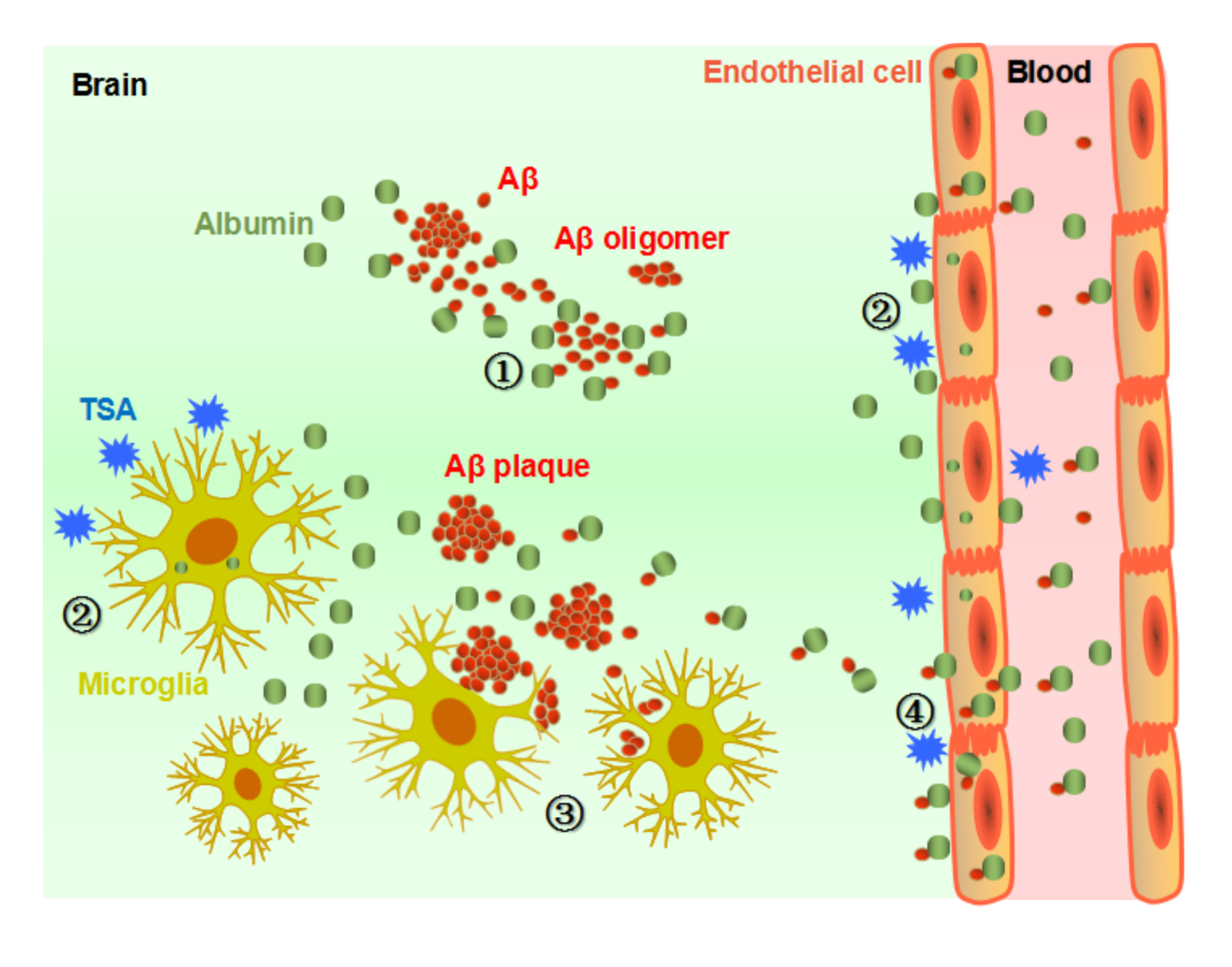
**
